# Sex-Specific Neuroplasticity in the Brain of a Facultatively Social Orchid Bee

**DOI:** 10.1093/icb/icag012

**Published:** 2026-03-23

**Authors:** Denise Yamhure-Ramírez, Marissa Sandoval, Santiago R Ramírez

**Affiliations:** Department of Evolution and Ecology, University of California, Davis, CA 95616, USA; Department of Evolution and Ecology, University of California, Davis, CA 95616, USA; Department of Evolution and Ecology, University of California, Davis, CA 95616, USA

## Abstract

Neuroplasticity enables the brain to reorganize in response to developmental change and experience, thereby supporting behavioral flexibility. In insects, both age and experience are known to influence neural structure, but how these factors differ between the sexes remains largely unexplored. Here, using micro-CT scanning, we quantify volumetric plasticity across eight brain regions in the orchid bee *Euglossa dilemma*, a facultatively social species with pronounced sexual dimorphism in behavior. We show that neuroplasticity follows sex- and region-specific trajectories that map onto the distinct reproductive behaviors exhibited by male and female bees. Consistent with other insect species, both sexes exhibited neuroplasticity in the mushroom bodies but only males showed an experience-dependent expansion, which we attribute to the navigational demands of reproductive behaviors. This was further supported by the expansion of olfactory and visual processing centers associated with the sensory demands of perfume collection and courtship display. Females, in contrast, undergo an exclusively age-dependent volumetric expansion of the mushroom bodies, an experience-dependent expansion of the antennal lobes, and a reduction of the visual neuropils. These patterns of plasticity correlate with female nesting behavior and may reveal potential energy trade-offs during reproduction. Our findings provide the first evidence of exclusively age-driven neuroplasticity in the mushroom bodies of social female bees and establish *E. dilemma* as a valuable comparative model for studying the evolution of brain plasticity and behavioral adaptation in social insects.

## Introduction

Neuroplasticity—the ability of the brain to remodel its structure and function in response to development and environmental conditions—is a widespread phenomenon across the animal tree of life (Lourenco and Casey 2013; Westwick and Rittschof 2021; Marzola et al. 2023). Across diverse animal taxa, neural circuits adjust to changing behavioral demands, allowing organisms to fine-tune sensory processing, learning, memory-formation, and decision-making as conditions change (Lourenco and Casey 2013). In insects, age- and experience-dependent changes in neuropil volumes have been documented across multiple taxa and are frequently linked to shifting cognitive demands, such as navigation, foraging, and social interactions (Groh and Meinertzhagen 2010). For example, the desert ant (*Cataglyphis bicolor*) exhibits enlargement of the mushroom body—a high-order processing center for associative learning and memory (Heisenberg 1998)—in older or navigation-active worker ants compared to younger or nest-bound colony members (Kühn-Bühlmann and Wehner 2006). In the harvester ant (*Messor pergandei*), mated queens exhibit reduced investment in the medulla—a visual processing center—after transitioning to dark subterranean nest environments (Julian and Gronenberg 2002). Together, these examples underscore how neuroplasticity facilitates behavioral flexibility and variation, thus providing insight into how neural investment aligns with the different ecological demands and conditions faced by individuals within a species.

Within bees, research in neuroplasticity has mostly focused on highly eusocial taxa such as honeybees and bumble bees, whose social organization and sensory ecologies are well described and understood (Withers et al. 1993; Winnington et al. 1996; Fahrbach et al. 1998; Jones et al. 2013; Marachlian et al. 2021). As a result, much of what is known and proposed in the models of bee neuroplasticity derives from such species, leaving facultative social and solitary lineages comparatively understudied (Withers et al. 2008; Smith et al. 2010; Rehan et al. 2015; Hagadorn et al. 2021b; Pahlke et al. 2021).

A second literature bias concerns sex. Even within eusocial taxa, research has focused on females, predominantly workers, given their key roles in colony maintenance and their practicality in numbers. In contrast, male bees have received very limited attention (Hagadorn et al. 2021a), leaving a critical gap in our understanding of how neuroplasticity evolves and varies within a species, particularly in relation to sex-specific and behavioral demands. Addressing this gap can provide key insight into how neural plasticity supports different behavioral repertories at the intraspecific level.

The facultatively social orchid bee *Euglossa dilemma* (Apidae: Euglossini) represents a particularly compelling system in which to investigate these questions. *Euglossa dilemma* is one of the most intensively studied orchid bee species, with detailed knowledge of its life history, ecology, and evolution (Brand et al. 2017; Saleh and Ramírez 2019; Darragh et al. 2023; Saleh et al. 2024; Yamhure-Ramírez et al. 2025). This species is considered facultatively social, as females exhibit a flexible social system in which a dominant mother, following an initial solitary phase, forms a small cooperative colony with one or two subordinate daughters.

Male bees emerge from the nest and fly long distances to collect volatile compounds used in courtship display (Dressler 1982; Pokorny et al. 2015; Brand et al. 2020). This behavior, which is central to the orchid bee reproductive biology, involves males actively gathering a diverse array of chemical compounds from various floral and non-floral sources to concoct a species-specific perfume used as a pheromone–analog (Henske et al. 2023). These collected fragrances are stored in specialized hind-leg pouches and later released during intricate display flights. Female bees are attracted to these chemical displays, which are thought to serve as indicators of male fitness and play a crucial role in mate selection. Through this process, the chemical composition and complexity of a male’s perfume influence his reproductive success by appealing to female preferences and thus facilitating mating success (Henske et al. 2023).

**Fig. 1:**
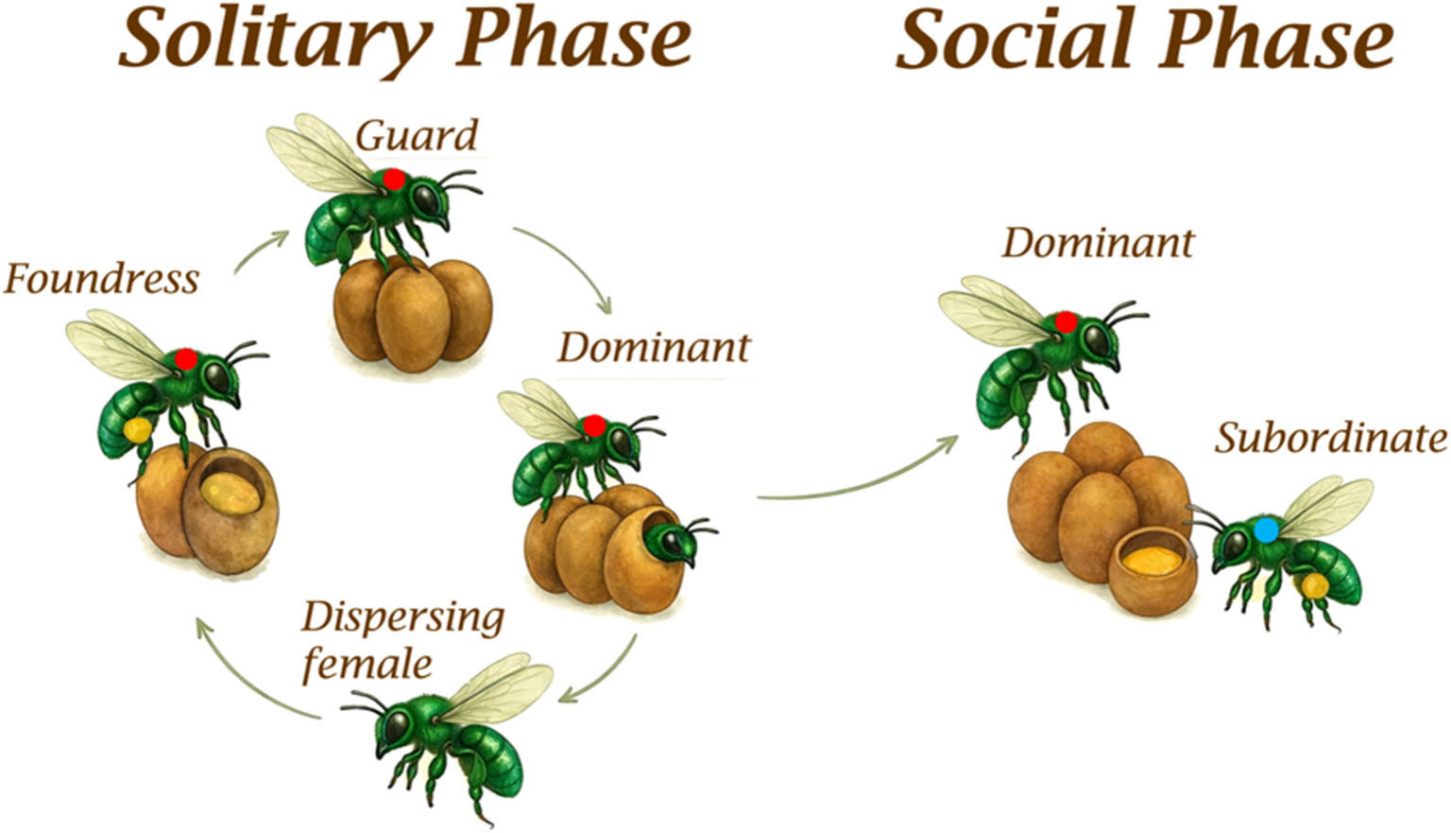
Life cycle of the female orchid bee, *E. dilemma*. Color tags follow individual bees through the cycle which begings with a dispersing female (no tag). Dispersing bees carry yellow pollen on the corbicula. A solitary foundress bee (red tag) initiates a nest and builds ∼6 brood cells. When a foundress completes the first batch of brood-cells, she stops foraging and transitions into a guard and remains inside the nest protecting the developing brood (4–6 weeks). Once offspring emerges, some female bees disperse to start new nests, while others stay inside the nest to become subordinate helpers (blue tag). Subordinates forage for resin and pollen, build brood cells, and lay eggs. Invariably, the dominant bee eats the eggs laid by the subordinate daughter and replaces them with her own, effectively controlling the reproductive output of the colony. Bee and brood cell images were generated with AI prompted with real images.

Females, in contrast, exhibit central place foraging, collect nesting materials like resin and pollen and exhibit social behavior inside the nests, which are established in pre-existing cavities (Saleh and Ramírez 2019; Brand et al. 2020; Saleh et al. 2021). The life cycle of a social nest consists of three distinct phases (Fig. 1) (Saleh and Ramírez 2019). In the first phase, a single mated female starts a nest as a foundress bee. After completion of the first batch of brood cells (on average 6–7 brood cells), she transitions to a guard phase, in which the female ceases foraging activity and brood cell construction, to remain inside the nest with a sealed entrance for up to two months until eclosion of offspring. The third phase begins when some of the emerged female offspring remain in the maternal nest to become subordinate helpers of the now dominant mother. The dominant mother, once the foundress bee, will maintain near-complete reproductive control by replacing the eggs laid by her subordinate daughters with her own eggs (Saleh and Ramírez 2019; Saleh et al. 2022, 2024).

The rich natural history of this orchid bee and its documented sexual dimorphism in behavior and ecological interactions provides a powerful system to disentangle how sex, age, and experience interact to shape the brain, while simultaneously elucidating how neural systems can be tuned to produce alternative adaptive strategies within a species.

In this study, we investigate how brain structures of the orchid bee *E. dilemma* change with age and experiences to test whether trajectories of neural plasticity differ between the sexes. Using high-resolution micro-CT imaging, we quantify volumetric investment across major brain regions in males and females of different ages and from distinct behavioral phases (i.e., foundress, guard, dominant, and subordinate). Specifically, we ask what brain regions exhibit age- or experience-dependent neuroplasticity between the sexes, and whether these changes map onto their distinct ecological and reproductive roles. Based on prior work, we predicted that experience-dependent neuroplasticity would be present in both male and female bees, although it would differ in magnitude particularly in the mushroom bodies, where it has been widely documented from solitary to eusocial species (Farris et al. 2001; O’Donnell et al. 2004; Kühn-Bühlmann and Wehner 2006; Molina and O’Donnell 2008; Withers et al. 2008; Hagadorn et al. 2021b). By comparing sex-specific plasticity with life-history transitions within a single species, our study provides a framework to investigate how neural systems evolve in response to behavioral and ecological adaptations.

## Materials and methods

### Field collections and experimental setup

For newly emerged bees, we sampled 13 females and 12 males that were sexed by the distinct morphology of their hindlegs. The data corresponding to these samples was used in a previous published study (Yamhure-Ramírez et al. 2025). In brief, wild *E. dilemma* nests with brood cells were collected in Florida from Flamingo Gardens, Fern Forest Nature Center, and Tree Tops Park. Nests were obtained following a previously established protocol that uses small wooden boxes (3.5 inches × 2.5 inches × 1.4 inches) with small entrance holes, which female bees colonize (Saleh and Ramírez 2019). Nests were shipped to UC Davis for daily monitoring and were kept at 25°C and under a 12/12 light-dark cycle for the controlled development of larvae/pupae. Nests were sealed and monitored daily to ensure bees were sampled during their first 36 h after emergence and had no interaction with the external environment.

To test for age-dependent neuroplasticity, nest boxes with brood cells were collected as indicated above. Collected nests were brought to the lab at the University of Florida Fort Lauderdale Research and Education Centre (FLREC) for daily morning monitoring of newly emerged bees. Nests were placed at 25°C under a 12/12 light-dark cycle and emerged bees were sexes as described above prior to starting treatment. Each individual bee was placed in a plastic container and maintained in isolation for 10 days with ad libitum food and without exposure to natural foraging, social interactions, or reproductive activity. Thus, this group represents a controlled age treatment independent of natural lifespan variation and social status to capture early post-eclosion brain maturation (Supplementary Fig. 1A). A total of 10 males and 11 females were collected from different nests, and for all samples, the treatment was initiated within the first 24 h after emergence.

To test for experience-dependent neuroplasticity, wild male bees were lured to a p-dimethoxybenzene scent bait and caught with a net. These males were then tested for relative experience by measuring the amount of perfume collected in their hind legs using Hirschmann 2 uL glass microcapillaries with an end-to-end system (Hirschmann Laborgeräte, Germany, Supplementary Fig. 1B). Only males with a full capillary (2 µL) load of perfume were collected and labelled as “experienced wild males.” Although this method is not a precise estimate of neither age or experience, it allowed us to set a lower-boundary threshold, as a full perfume load takes at least 3 weeks to be accumulated (Henske et al. 2023). During these 3 weeks, males are likely to acquire experience during foraging for nectar and perfume compounds, male–male interactions, courtship display, and perfume exposure. Sampling was carried in the above-mentioned nature reserves, and 13 males were collected.

Wild-caught females were selected based on social status in the nest, which we determined by marking individuals and monitoring their behavior for at least 1 week using continuous video recordings. Individual bees were classified following established criteria of discrete behavioral traits for this species, which included foraging times and social interactions (Saleh and Ramírez 2019). We sampled bees in the guard and dominant phases, which represent mated females with reproductive potential and constitute successive behavioral phases (Fig. 1). All the guard bees we sampled had a minimum of seven brood cells, thus establishing a lower threshold of foraging experience. Dominant bees were sampled from social nests with at least one subordinate daughter. Neither subordinate bees nor foundress bees were included in this study due to the difficulty in obtaining enough replicates for these two groups. Our sampling approach thus allowed us to compare wild-caught females with consecutive behavioral phases that provide age structure in our comparative analysis. A total of 10 guards and 9 dominant bees were analyzed, all of which were collected from nests at Flaming gardens and the FLREC pavilion. The complete behavioral cycle is shown in Fig. 1.

We note that our design uses a 10-day treatment to capture early post-eclosion brain remodeling under controlled conditions, representing age-driven neuroplasticity, whereas wild-caught individuals capture the cumulative effects of age and experience under natural conditions. Thus, our design does not rule out possible subsequent changes caused by age alone in older bees but rather disentangles early brain growth due to age alone from changes that likely reflect neural remodeling caused by the cumulative effects of age, foraging, navigation, reproductive behaviors, and social interactions. A summary table of the sampled groups can be found in Supplementary Table 1.

### Sample preparation and image processing

All sampled bees followed the same sample preparation for micro-CT scanning published by Yamhure-Ramirez et al. (2025). Briefly, bees were anesthetized in cold, followed by immediate decapitation and overnight fixation in formaldehyde alcoholic acetic acid. After fixation, samples were individually stained in a 1% iodine solution in 100% ethanol made using resublimed iodine crystals (Spectrum Chemical Mfg. Corp, New Jersey) for 48 h at room temperature in the dark. Samples were then washed twice for 30 seconds in 100% ethanol and stored in 100% ethanol.

Image acquisition and processing replicated a previously published protocol (Yamhure-Ramírez et al. 2025). In brief, scans were obtained at the Center for Molecular and Genomic Imaging (CMGI), University of California, Davis, USA, using a MicroXCT-200 CT specimen scanner (Carl Zeiss X-ray Microscopy Inc., Pleasanton, USA). Samples were submerged in 100% ethanol while scanned with a 4.0X objective and 3200 projections per sample, achieving a 5.24 μm voxel size resolution. Image reconstruction was done using *Zeiss XM Reconstructor* software, which applied a smoothing filter of 0.5 kernel size to decrease image noise. Each reconstructed sample was explored as an individual 16 bit-raw tiff for posterior analysis using the free academic license of the Dragonfly 3D World software, Version 2024.1 for Windows. Comet Technologies Canada Inc., Montreal, Canada; software available at https://www.theobjects.com/dragonfly.

All tiff files were imported to Dragonfly ORS for image processing and segmentation of individual brain volumes (µm^3^). For a correct visualization of the brain, scans were re-oriented in the 2D views (XY, XZ, YZ) using 6 degrees of freedom to achieve natural axis-orientation. For each sex, one bee scan was used to register and align all other scans to conserve axis orientation throughout all the tested groups. We tested for neuroplasticity in eight main regions of the brain: lamina, medulla, lobula, antennal lobes, lateral horn, central complex, mushroom bodies, and the anterior optic tubercle. The lower unit of the anterior optic tubercle (AOTu) and what can be the start of the anterior optic tubercle tract were not distinguishable with the scan’s resolution. We preformed segmentation in all scans equally, segmenting until the contrast line connecting to the upper unit of the AOTu was no longer visible (Supplementary Fig. 2). Each neuropil was isolated as an anatomical independent structure based on the brightness of the iodine staining and following the delimitation used in Yamhure-Ramírez et al. (2025) to allow for consistency during multiple comparisons.

The central complex was analyzed as one single structure including the protocerebral bridge, fan-shaped body, ellipsoid body, and the noduli, maintaining the same approach from (Yamhure-Ramírez et al. 2025). The mushroom bodies were tested as one single structure, without differentiating between the peduncles and the calyx, as here we aim to provide a first broad understanding of the overall neuroplasticity changes occurring across the brain. Segmentation of the calyx subcompartments (lip, collar, and basal ring) was not performed in this study, as some scans differentiation of these regions proved challenging and we opted for a conservative approach. We also omitted the segmentation and analysis of Kenyon cell volumes, which we previously showed to be larger in newly emerged females than males (Yamhure-Ramírez et al. 2025). We decided to omit this measurement in the present study as using volume as a proxy of cell number assumes equal soma size and density, which can mislead interpretations.

For each scan, selected brain areas were segmented as isolated three-dimensional regions of interest (ROIs) using manual and semi-automatic tools. A smoothing filter of 7 points kernel size was applied to each ROI to match the standardization of noise reduction with that of newly emerged bees. For each neuropil, the total volume (μm^3^) was finally extracted from the statistical properties panel. In addition to the eight selected brain regions, we additionally measured the volume of the remaining central brain (rCB) as an independent measure of brain size to perform statistical analyses controlling for allometric scaling (Montgomery et al. 2016).

### Statistical analysis

Statistical analyses were performed in RStudio 2025.05.1 “Mariposa Orchid” Release (ab7c1bc795c7dcff8f26215b832a3649a19fc16c, 2025–06-01) for windows using R version 4.4.3. All analyses were performed using the log_10_-transformed value of the absolute volume of each ROI.

Prior to testing neuroplasticity changes driven by either age or experience, we used the package smatr v.3.4–8 (Warton 2006) to run standardized major axis regressions using the allometric scaling relationship: log *y* = β log *x* + α and the smart function sma(y∼x*groups) to confirm no significant difference in the allometric scaling of each ROI to the rCB. We expected no differences in scaling given our previous results (Yamhure-Ramírez et al. 2025). After confirming a shared allometric scaling across groups, both within and between sexes, we tested for grade-shifts along the *Y*-axis. Significant deviations from the alpha value of newly emerged bees indicated changes driven by neuroplasticity within each sex and further allowed us to compare the strength of such changes between sexes.

For each ROI, we performed multiple comparisons that included all male and female groups with the smart package function sma(y∼x + groups). All regressions were run using the argument robust = T, multcomp = T, and multcompmethod=“adjust” to account for low sample size and control the probability of false-positives by using adjusted *P*-values via the “Sidak adjustment” (Westfall et al. 1993) applied by this function. All final plots were created using the *ggsmatr* R package Sandoval, M. (2025). *ggsmatr* (Version 1.0) [Source code]. GitHub. https://github.com/mariosandovalmx/ggsmatr.

To identify relative expansions of different brain regions with significant deviations from newly emerged bees, we used the direct proportionality rule, which allows us to find any missing value if $x1:x2 = y1:y2$, thus equivalently ${{y}_2} = ( {{{x}_2} \times {{y}_1}} )/{{x}_1}$.The log values of the y-intercepts (α) were converted to their corresponding integer value (x1 and y1), after which the relative expansion of a region was calculated by setting the α value of newly emerged bees to 1 delimiting the baseline for the proportional values of each brain region. For example, after applying the proportionality rule to a region with significant differences (e.g., mushroom bodies), a value of ${{y}_2}\ $=1.15 corresponds to a 15% expansion driven by neuroplasticity. A summary of the calculated expansions can be found in Supplementary Table 2.

## Results

In both sexes of *E. dilemma*, age and experience-dependent neuroplasticity are differentially distributed across the brain, showing region- and sex-specific responses in all but the central complex.

We note that in our results, the variance differs between brain regions and across sexes, particularly on wild-caught bees. This increased variability likely reflects biological heterogeneity among wild-caught individuals. While our sampling methodology was sufficient to establish a lower-bound threshold for age and experience; however, given the long lifespan of this species, the individuals collected could range in navigation, foraging experience, reproductive behaviors, and social interactions, all of which likely contribute to the variance around the mean.

### Volumetric changes in the mushroom body

**Fig. 2:**
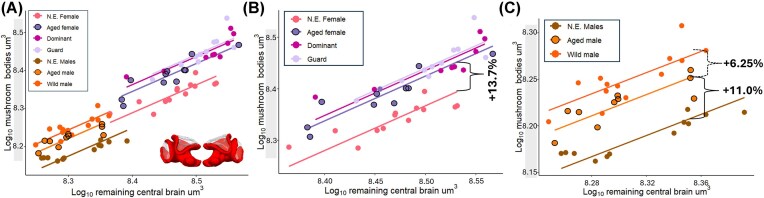
Sex-specific age- and experience-dependent plasticity of the mushroom bodies relative to remaining central brain volume. Females: newly emerged (N.E.), aged (laboratory-reared), dominant, and guard. Males: newly emerged, aged (laboratory-reared), and wild (free-flying) (**A**) Log₁₀-transformed mushroom body volume (µm³) plotted against Log₁₀-transformed remaining central brain volume (µm³) across groups of female and male bees. Each point represents one individual bee. Lines depict least-squares linear regressions for each group. Across sexes, age-related mushroom body expansion in males eliminates the sexual dimorphism present at emergence (newly emerged females vs. newly emerged males: *P* = 1.22–15; newly emerged females vs. aged males: *P* = 1.0; aged males vs. aged females: *P* = 0.97). (**B**) Female-only regressions illustrating age but not experience effects. Relative to newly emerged females, aged females exhibit a 13.7% increase in mushroom body (*P* = 5.4 × 10⁻⁹). No additional experience-dependent expansion was detected among social roles (dominant, guard). Solid curly brace indicates age-driven expansion. (**C**) Male-only regressions illustrating age and experience effects. Aged males show an 11.0% increase in mushroom body volume relative to newly emerged males (*P* = 5.29 × 10⁻¹³). Wild males exhibit a secondary expansion of 6.25% compared to aged laboratory males, indicating experience-dependent neuroplasticity (*P* = 0.04). Solid curly brace indicates age-driven expansion; dashed curly brace secondary expansion.

The mushroom bodies were the only brain region that exhibited age-dependent plasticity in both male and female bees (Fig. 2A). In females, this neuropil increased with age by an average of 13.7% relative to its original size (*P* = 5.4 × 10⁻⁹, test statistic = 39.97), with no evidence of further expansion driven by experience (Fig. 2B). Aged males responded similarly, showing 11% increase in volume (*P* = 5.29 × 10⁻¹³, test statistic = 58.07), however, in males, we found evidence of a secondary volumetric expansion, accounting for an additional 6.25% increase in size (*P* = 0.04, test statistic = 9.55) driven by experience (Fig. 2C).

Comparisons between the sexes showed that with the initial age-driven volumetric expansion in males, the volume of the mushroom bodies is comparable to those of aged, guard, and dominant bees (*P* > 1 for every pair-wise comparison). This expansion, thus eliminates the previously reported dimorphism observed in newly emerged bees (Yamhure-Ramírez et al. 2025).

### Volumetric changes in olfactory neuropils

**Fig. 3:**
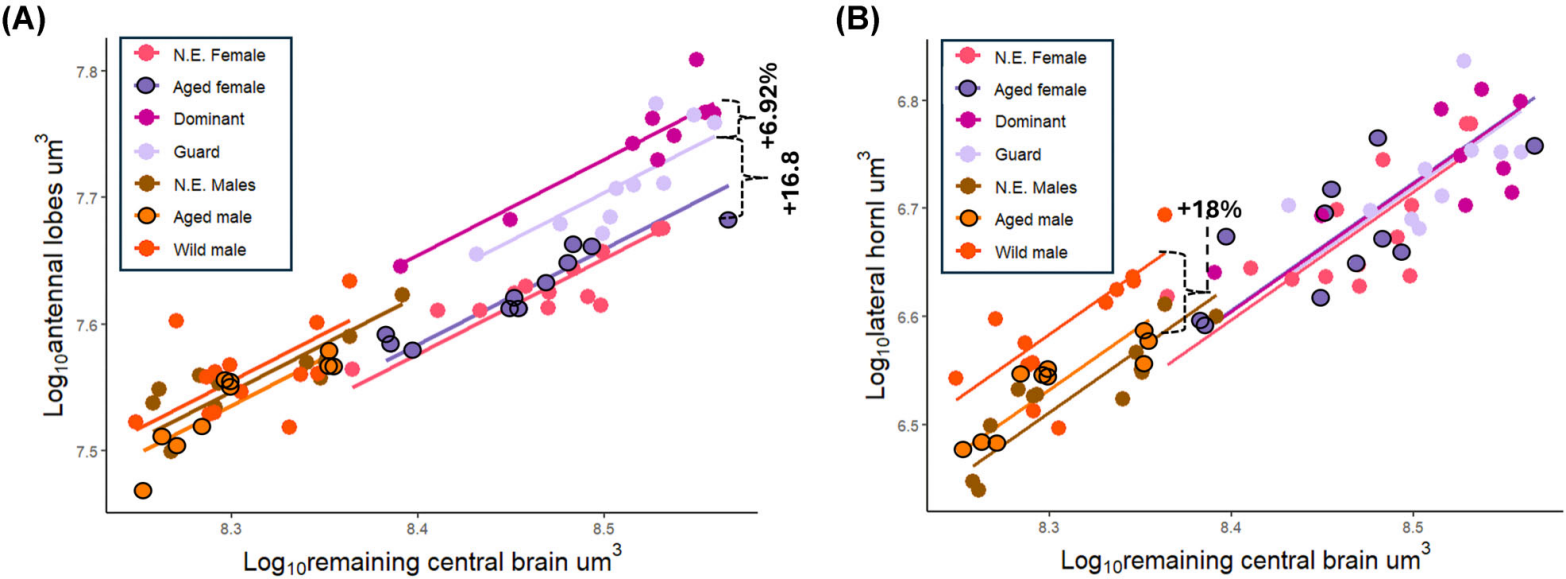
Sex-specific experience-dependent plasticity in olfactory neuropils relative to remaining central brain volume. Females: newly emerged (N.E.), aged (laboratory-reared), dominant, and guard. Males: newly emerged, aged (laboratory-reared), and wild (free-flying). (**A**) Log₁₀-transformed antennal lobe volume (µm³) plotted against Log₁₀-transformed remaining central brain volume (µm³) across groups of female and male bees. Lines indicate least-squares linear regressions for each group. In females, antennal lobe volume increases progressively with experience, with guard and dominant individuals exhibiting cumulative expansion relative to newly emerged and aged females, consistent with experience-dependent plasticity independent of age (newly emerged females vs. guards: *P* = 0.0001; newly emerged females vs. dominants: *P* = <2.22e-16, guards vs. dominants: *P* = 0.01). No significant volumetric differences were detected among male groups at the whole-antennal-lobe level. Dashed curly braces represent cumulative significant expansions. (**B**) Log₁₀-transformed lateral horn volume (µm³) plotted against Log₁₀-transformed remaining central brain volume (µm³). Symbols and regression lines as in (**A**). In contrast to females, males exhibit experience-dependent expansion of the lateral horn: wild males show a significant increase in volume relative to newly emerged and aged laboratory males (*P* = newly emerged male vs. wild male: *P* = 1.42e-05), whereas no significant age-dependent differences were detected between newly emerged and aged males. Females show no significant volumetric changes in the lateral horn.

Neuroplasticity in the antennal lobe—the primary olfactory processing center—was restricted to female bees. No evidence of significant changes among male groups was observed (Fig. 3A). Amongst females, volumetric expansion was only seen within experienced bees (guard and dominant, Fig 1), with age seemingly having no effect (*P* = 0.29, test statistic = 1.13). Both guard and dominant bees had a significant volumetric increase compared to newly emerged females (Guards: *P* = 0.0001, test statistic = 20.2; Dominants: *P* = <2.22e-16, test statistic = 89.7). Guarding bees showed a 16.8% increase in their antennal lobe volume, with dominant bees showing a secondary significant enlargement of 6.92% compared to guarding bees (*P* = 0.01, test statistic = 6.09).

In wild-caught males, the lateral horn, a secondary processing center for olfactory information of learned and innate olfactory responses, showed evidence of volumetric expansion (*P* = 1.42e-05, test statistic = 1.88e + 0) with an 18% increase in size relative to newly emerged males (Fig. 3B).

### Volumetric changes in visual neuropils

In bees, the primary visual processing center known as the optic lobe is comprised of three nested neuropils: the lamina, medulla, and lobula. These three neuropils are located beneath the photoreceptive retina, and each region processes different types of visual information. The lamina is known to be involved in contour delimitation through dark–light contrast (Douglass and Strausfeld 2004). The medulla processes spectral opponency—a mechanism by which color is encoded through opposing neural responses (Lee et al. 2002; Paulk et al. 2009a, et al. 2009). The lobula is mainly involved in object recognition and motion detection (Paulk et al. 2009b; Strausfeld 2009; Hempel de Ibarra et al. 2014).

We found substantial neuroplasticity in the optic lobe exclusively in guard and dominant female bees, both of which spend most their time inside the nest, with the lamina and the medulla showing a significant reduction in their relative volume relative to newly emerged and aged bees. The lamina, on average, decreased by 25.9% of its original size (lamina: *P* = < 2.11e-11, test-statistic = 4.49 in guards; *P* ≤ 2.22e-16, test-statistic = 1.48 in dominants). The medulla, on average, decreases 14.5% (medulla: *P* = 2.01e-07, test-statistic = 27.03 in guards; *P* = < 2.22e-16, test-statistic = 70.6 in dominants).

**Fig. 4:**
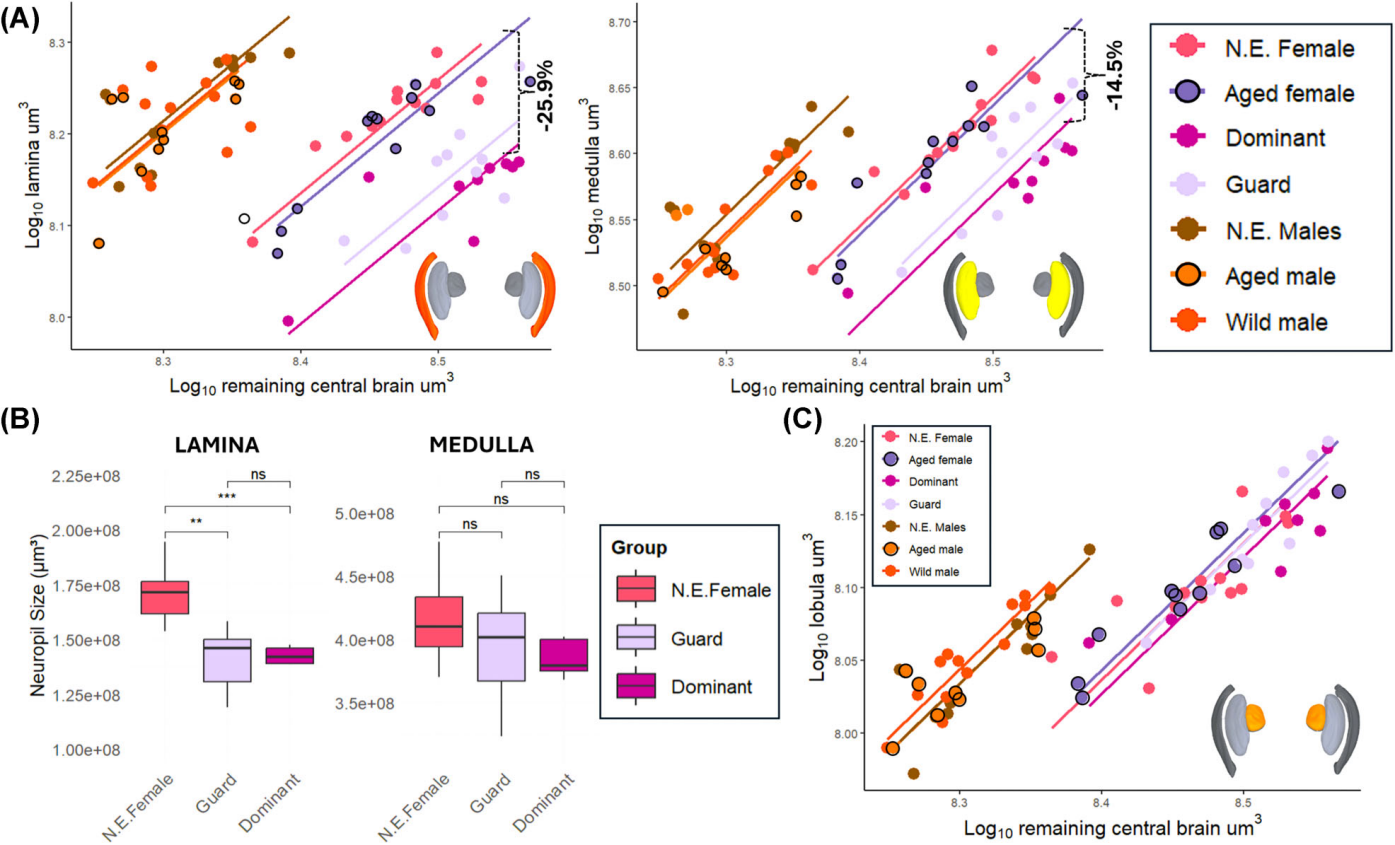
Sex-specific effects of experience-dependent neuroplasticity on the optic lobe. (**A**) Guard and dominant female bees show a reduction in the size of the lamina (orange color-coded) and medulla (bright yellow color-coded) indicating experience-dependent changes (dashed curly brace) in the two outmost neuropils of the optic lobe. (**B**) Neuroplasticity in the absolute volumes of the lamina and medulla. The lamina shows a significant reduction in absolute size between newly emerged and experienced guard and dominant bees, but these effects were not observed in the medulla. Boxes indicate interquartile range, lines are medians, and whiskers extend to 1.5 the interquartile range. Wilcoxon signed-rank test: **P = 0.01; ***P = 0.001. (**C**) The lobula (mustard yellow color-coded), the inner most neuropil of the optic lobe, shows no neuroplasticity with each regression line following the trend of the newly emerged bees within each sex.

When evaluating differences in the absolute volumes of these regions, the lamina of guard and dominant bees was 16.1% smaller than in newly emerged female bees (*P* = 4.80 × 10⁻^4^). In the medulla, no significant changes in absolute volume were observed, yet we found a tendency toward significance (*P* = 0.08), with an average size 5.5% smaller than newly emerged bees (Fig. 4B). In the lobula, the innermost neuropil, we found no evidence of volumetric change in either of the sexes (Fig. 4C).

In addition to the optic lobes, the anterior optic tubercule—a secondary processing center of visual information—showed evidence of experience-dependent neuroplasticity with a volumetric expansion in both sexes (Supplementary Fig. 3). This expansion accounted for an increase of 22.90% in wild males, and an average increase of 13.5% across guard and dominant bees, which showed no significant difference among them.

## Discussion

Our results reveal a pattern of neuroplasticity that follows sex- and region-specific trajectories that correlate with the distinct ecology and behavior of male and female bees of *E. dilemma*. By disentangling the independent effects of age and experience on volumetric changes across eight major neuropils, we show that both sexes exhibit age-related expansion confined to the mushroom bodies, whereas experience-dependent changes were observed across different brain regions in a distinct, sex-specific pattern.

### Sex-specific patterns of mushroom body expansion

Our results demonstrate that age, and not experience, is the primary driver of the change in the mushroom bodies in both sexes (Fig. 2A), accounting for a volumetric increase of 13% in females (Fig. 2B) and 11% in males (Fig. 2C). In contrast, experience only contributed to a secondary and less pronounced expansion (6.2%) in males (Fig. 2C). To our knowledge, this provides the first evidence of exclusively age-dependent neuroplasticity in females of a social bee.

Across Hymenoptera, experience-dependent neuroplasticity has been well documented in both social and solitary species as a mechanism supporting spatial navigation, memory consolidation, and higher-order integration (Withers et al. 1993; Kühn‐Bühlmann and Wehner 2006; Withers et al. 2008a; Hagadorn et al. 2021b). In solitary and facultatively social bees—such as *Osmia lignaria* (solitary) and *Ceratina calcarata* (facultatively social)—the mushroom bodies show cumulative neural expansion following exposure to novel environments, floral resources, and social interactions, compared to newly emerged individuals (Withers et al. 2008a; Rehan et al. 2015). Similarly, in eusocial species, the onset of foraging experience and/or social interactions can contribute to more pronounced neuroplasticity in the mushroom bodies after age-related expansions have occurred early in adult life (Fahrbach et al. 1998; Farris et al. 2001; Riveros and Gronenberg 2010; Jones et al. 2013; Tomé et al. 2014; Cabirol et al. 2018). Most studies of honeybees use the single-cohort method to compare nurse bees and precocious foragers of the same age (10–11 days), which defers from our experimental design. However, Maleszka et al. showed that in isolated honeybees mushroom bodies expanded only slightly with age, whereas foragers exhibited the most significant expansion regardless of age when compared to age-matched hive bees.

Age-related neuroplasticity has been especially well documented in social species and is often interpreted as a shift toward pre-programmed neural adaptations for predictable life history events or transitions. Examples include pronounced changes in brain volumes of honey bee workers (Fahrbach et al. 1998; Farris et al. 2001), stingless bees (Tomé et al. 2014), bumblebees (Riveros and Gronenberg 2010), facultatively eusocial sweat bees (Hagadorn et al. 2021a), and wasps (O’Donnell et al. 2004; Molina and O’Donnell 2008). In solitary taxa, however, age-dependent neuroplasticity appears to be absent, for which it has been proposed as a mechanism coupled with social evolution (Withers et al. 2008; Hagadorn et al. 2021b). Our results show age-related plasticity in males and age-limited plasticity in females of a facultatively social species. Alongside these findings, this positions *E. dilemma* as an informative comparative system to further investigate age-related plasticity in the context of social evolution.

Few studies of solitary and facultatively social bees have incorporated an isolated age treatment (Jaumann et al. 2019; Hagadorn et al. 2021b), limiting direct comparisons of age-dependent and experience-dependent neuroplasticity across species. However, in this study, the predominance of age over experience as the main driver of the mushroom body expansion may represent a distinctive aspect of orchid bees best explained by their life history.

Our results suggest that in females of *E. dilemma* age-dependent neuroplasticity of the mushroom bodies is a canalized neurodevelopmental process that supports the behavioral flexibility of newly emerged female bees. These bees can become solitary foundresses or subordinate helpers, but both groups exhibit similar behaviors such as foraging for resin and pollen to build brood cells (Saleh et al. 2022). Because both solitary foundresses and subordinate helpers must perform comparable foraging and nest-building activities, selection may have favored a robust, age-dependent maturation of the mushroom bodies that buffers against ecological variability while supporting the flexible social roles of *E. dilemma* females. Interspecific comparative studies between facultatively social bees that incorporate an explicit “age in a cage” treatment can provide a valuable model for exploring how social complexity influences neural plasticity during evolutionary transitions.

Our results also suggest that, in *E. dilemma* males (solitary), the predominantly age-dependent neuroplasticity may reflect an adaptive developmental programming aligned with the orchid bee’s mating ecology. Male orchid bees engage in highly specialized behaviors soon after emergence, including long-distance flight to concoct an extended phonotype perfume used in courtship displays. These reproductive behaviors impose substantial sensory integration and cognitive demands that may be developmentally anticipated rather than driven by experience. This might also explain why the age-driven expansion of the mushroom bodies in males is enough to match the size of this neuropil across female groups.

The secondary expansion of the mushroom bodies seen in wild-caught males is likely linked to experience, particularly the cognitive demands associated with male reproductive biology. In Euglossini bees, male reproductive success strongly relies on their capacity to concoct and expose their pheromone–analog perfume (Henske et al. 2023). Compared to males of other bee species, which typically engage primarily in mate searching or patrolling behaviors, male Euglossini bees require extensive foraging, orientation flights, territory patrol, volatile collection, and potential spatial learning of display sites for mating success (Dressler 1982; Stern 1991; Pokorny et al. 2015; Henske et al. 2023). This combination of behaviors might impose unusually high integrative demands on learning and memory circuits. Such experience-dependent neuroplasticity is consistent with findings in other Hymenoptera in which male neural investment is tightly coupled to reproductive strategies (Molina and O’Donnell 2008). However, because the “aged” group was at most half the age of the experienced wild-caught males, the current experimental design cannot rule out the possibility of this secondary expansion being age-dependent.

Our results highlight the importance of cognitive pressures that arise from both social interactions and reproductive demands in both sexes. Future research should investigate whether these volumetric changes correspond to the expansion of similar or different neural circuits between sexes to better inform the functionality of these expansions.

### Divergent trajectories in the expansion of olfactory neuropils

We identified clear sex-specific differences in the plasticity of the olfactory neuropils, specifically the antennal lobes and lateral horn, which may reflect differences in the type of olfactory information processed and its associated behavioral outcomes.

In experienced female bees, the antennal lobes expand progressively in guard and dominant bees (Fig 3A), correlating with an increased demand for novel and learned olfactory cues related to nest recognition, kin interactions, and reproductive dominance (Saleh and Ramírez 2019). This expansion aligns with previous evidence of antennal lobe plasticity, which requires direct sensory input associated with social, reproductive, and/or foraging demands with complete independence from age. (Winnington et al. 1996; Jones et al. 2013; Eriksson et al. 2019). In contrast to the plasticity observed in females, we found that the male antennal lobes exhibit no volumetric expansion. We note that the lack of reported neuroplasticity in males at the whole-antennal-lobe level does not imply that changes at a finer structural level are absent. Potential glomerulus-specific changes may have gone undetected and merit further investigation. Instead, we found pronounced plasticity in the lateral horn (Fig. 3B), a brain region that is involved in modulating various types of innate behaviors via neural circuits that encode biological valence of novel odor cues (Schultzhaus et al. 2017).

The male-specific expansion observed in the lateral horn likely reflects the integration and valance of diverse volatile compounds acquired by male bees. In *Drosophila*, innate responses to odors, including sex pheromones, has been linked to circuits in the lateral horn (Schultzhaus et al. 2017). We speculate that the plasticity we observed in the lateral horn in *E. dilemma* males corresponds to the acquisition and collection of perfume compounds, which is a key aspect of their reproductive biology. Male orchid bees spend much of their life collecting specific perfume compounds from the environment to use in courtship display. The collected compounds, which are stored in enlarged specialized hindleg pouches, are later exposed during courtship display and thus act as pheromone–analogs directly linked to mating success (Henske et al. 2023). Perfumes are highly species-specific and show little variation within a species across geography and seasons (Zimmermann et al. 2009; Darragh et al. 2023) and thus this behavioral trait is likely to be driven by innate circuits. However, controlled experiments are required to validate these results.

### Remodeling of visual regions is associated with energy trade-offs and navigational demands

In females, but not males, we found a pronounced volumetric reduction in the lamina and medulla of guard and dominant bees (Fig 4A). The absence of change in both aged female bees and across all male groups is likely to indicate the reported volumetric reductions in these regions are associated with the transition to these two distinct social phases (Fig. 4A), known to be accompanied by behavioral and physiological changes, as well as differential gene expression in the ovary and brain (Saleh and Ramírez 2019; Saleh et al. 2022). These results correlate with loss of active foraging and habituation to a dark environment, echoing findings from queen ants and king termites, which show a significant decrease in the medulla after transitioning to nest-bound lifestyles (Julian and Gronenberg 2002; Ishibashi et al. 2023).

The lamina and medulla are brain regions involved in contrast enhancement and color vision processing (Hempel de Ibarra et al. 2014; Ketkar et al. 2022), which are sensory demands expected to decrease with the transition to guard and dominant behavioral phases. As maintaining neural tissue is metabolically costly (Niven and Laughlin 2008), we propose that in *E. dilemma* this reduction may act as a mechanism to optimize energy that will be needed for reproductive roles. This idea aligns with the energy trade-off hypothesis, an extension of the expensive tissue hypothesis (Aiello and Wheeler 1995) which argues that the body allocates its metabolic energy between competing energy-consuming functions.

The absence of change in both aged female bees and across all male groups is likely to indicate the reported volumetric reductions in these regions are associated with the transition to the guard and dominant social phases (Fig. 4A). The transition to these phases is also known to be accompanied by behavioral and physiological changes, as well as differential gene expression in the ovary and brain (Saleh and Ramírez 2019; Saleh et al. 2022).

Interestingly, the lobula, the innermost neuropil of the optic lobe, showed no volumetric change among female behavior groups (Fig 4B), suggestive of either ongoing functional demands or a lack of plasticity potential via phenotypic stability despite environmental variation. This region is known to play a role in shape discrimination, motion detection of small objects darker than the background and processing of optic flow (O’Carroll 1993; Keleş and Frye 2017; Erginkaya et al. 2025), with additional evidence showing its responses can be modulated by odor influx (Vinauger et al. 2019). These observations may reflect visual demands needed for both in-nest interactions with brood cells and subordinate daughters, as well as stabilizing vision and steering during flight (optic flow), as guard and dominant bees continue to forage for nectar with short flights at least once a day. The lobula is also known to contribute to figure–ground segregation, the ability to discriminate a moving object from its background through temporal coherence, and to relay object motion signals to the anterior optic tubercle (Bockhorst and Homberg 2017; Omoto et al. 2017). Intriguingly, despite no volumetric change in the lobula, the anterior optic tubercle showed an expansion of 13.5% in guard and dominant bees (Supplementary Fig 3).

The observed divergent patterns of neuroplasticity in the optic lobe—namely, the reduction in the lamina and medulla but stability in the lobula—are indicative of a selective reorganization of visual processing centers in *E. dilemma* females. These results may be explained by the maintenance of visual circuits that support in-nest behaviors such as social interactions and nest maintenance, while reducing energy expenditure by downsizing sensory processing areas with decreased demands. We note that the lack of observed changes in the lobula does not rule out changes at the circuit level.

We also identified an expansion of the anterior optic tubercle in wild-caught males that accounted for a pronounced volumetric increase of 22.9% (Supplementary Fig. 3). This expansion likely reflects increased investment in neural circuits underlying spatial navigation needed for reproductive success. A similar explanation has been proposed for the relative enlargement of the optic lobes of males bees (Barrett et al. 2021), and the morphological integration with the central complex in *E. dilemma* (Yamhure-Ramírez et al. 2025).

Across insect taxa, directional orientation is mediated through a conserved visual pathway. Visual input from the optic lobe, specifically the medulla and lobula, convey information to the anterior optic tubercule, which acts as a major relay center to the central complex for sky-compass navigation and motor planning (Träger et al. 2008; Mota et al. 2011). Thus, the expansion of this neuropil in males aligns with their reproductive behavior, in which males repeatedly visit perfume sources and use the same perch sites across multiple days and weeks during courtship display (Henske et al. 2023), a behavior that requires compass-based navigation.

Intriguingly, the central complex did not differ in overall volume across groups; however, this does not preclude the possibility of expansion within its individual subregions. In ants, color learning and memory formation have been shown to increase the volume of the fan-shaped body and protocerebral bridge, while the ellipsoid body and noduli remain unchanged (Yilmaz et al. 2019). As with the antennal lobe, examining these substructures individually may therefore warrant further investigation. Our study highlights the role of neuroplasticity as a dynamic mechanism that supports the complex sex-specific behaviors observed in insects. Female bees exhibit a pronounced remodeling of visual and olfactory centers across reproductive and social transitions that suggest the existence of energetic trade-offs that correlate with their life history. Males, on the other hand, exhibit changes in neural regions that are known to support innate processing of olfactory signals (e.g., perfume acquisition), and navigation (e.g., courtship display). We note that controlled experiments are needed to test whether the observed neuroplasticity is itself is a sexually dimorphic adaptation, or if this capacity is shared across sexes but contingent to the sensory and cognitive demands an individual experiences. This could help elucidate the genetic basis of neuroplasticity at the species level.

Future work should identify fine-scale volumetric changes (e.g., glomeruli of the male antennal lobe) to determine whether the results reported here correspond to specific stimuli and whether they extend to other facultative social species, thereby clarifying the neural mechanisms underlying the evolution of social and mating behaviors.

## Supplementary Material

icag012_Supplemental_Files

## Data Availability

The findings of this study are supported by data provided in the supplementary information files, and files available in the Figshare repository under the following link: https://figshare.com/s/95006b8e7b6bf0f36be8.
