# Supplementary material for: Sex-Specific Neuroplasticity in the Brain of a Facultatively Social Orchid Bee
Source: Integr Comp Biol. 2026 Mar 23;66:icag012. doi: 10.1093/icb/icag012 (PMC13069684; doi:10.1093/icb/icag012)
Supplement: icag012_Supplemental_Files [file icag012_supplemental_files.zip › icb-2026-0003-File007.docx]

**Supplementary Material- Sex-specific neuroplasticity in the brain of a facultatively social orchid bee**


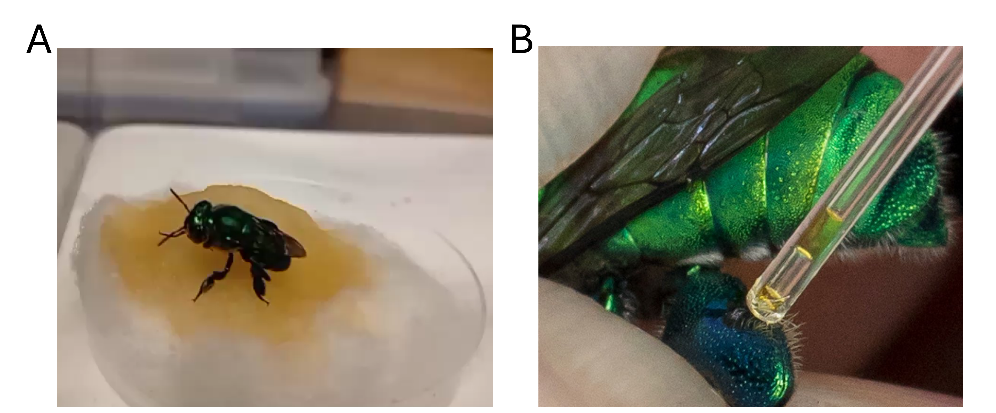


**Supplementary Fig 1** Graphic summary of methodology employed for groups characterization. A) Photo of age treatment taken by Denise Yamhure-R. B) Photo of male perfume collection taken by Marissa Sandoval.


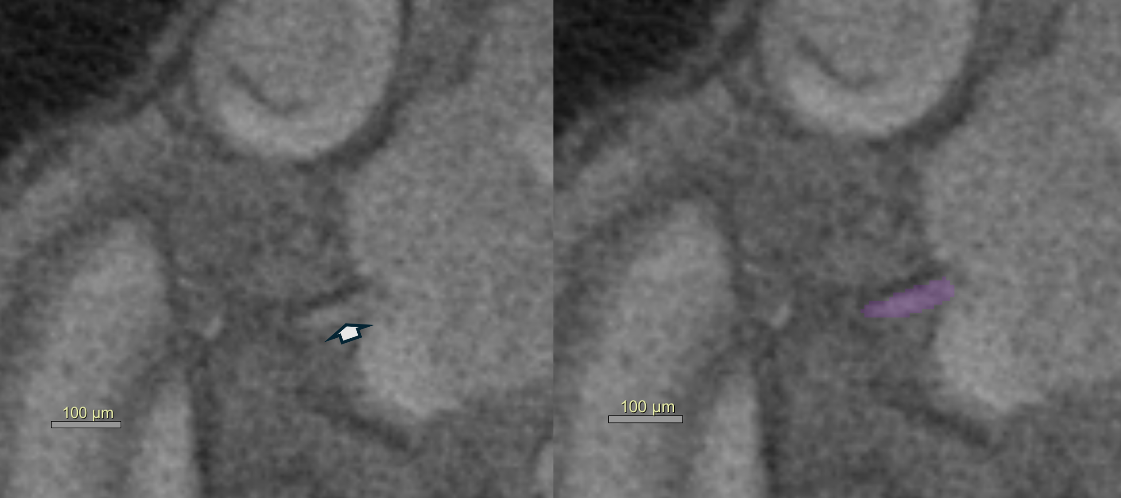


**Supplementary Fig 2. Segmentation of the anterior optic tubercle. The scans show the point at which the segmentation stopped, delimited by subsequent lack of contrast of the line connecting to the upper unit of the anterior optic tubercle.**

**
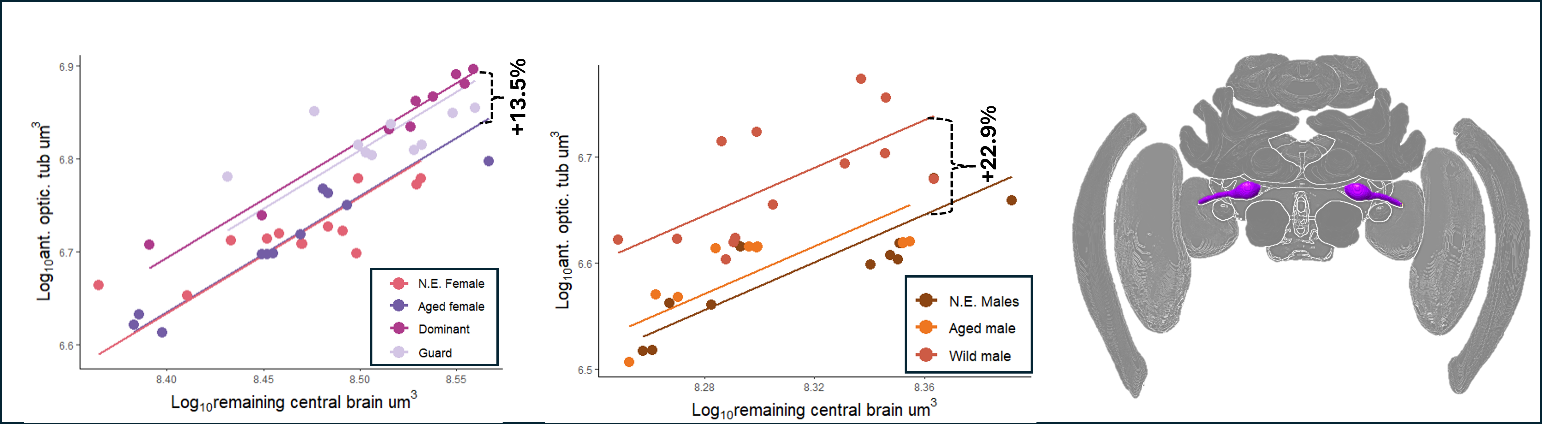
**

**Supplementary Fig 3**- The scale of experience-dependent neuroplasticity in the anterior optic tubercule (color coded in violet) differs among sexes.
