# Supplementary material for: Sex-Specific Neuroplasticity in the Brain of a Facultatively Social Orchid Bee
Source: Integr Comp Biol. 2026 Mar 23;66:icag012. doi: 10.1093/icb/icag012 (PMC13069684; doi:10.1093/icb/icag012)
Supplement: icag012_Supplemental_Files [file icag012_supplemental_files.zip › icb-2026-0003-File008.docx]

**Supplementary Table 1. Treatment Groups**

| Group Name | Age Class | Experience | Sample Size (n) |
| --- | --- | --- | --- |
| Newly Emerged Female | < 36 hours | No | 13 |
| Aged Female (Lab) | 10 days | No | 11 |
| Guard Female | > 4 weeks ***** | Yes: Mating, nesting, foraging and guarding | 10 |
| Dominant Female | > 8 weeks ***** | Yes: Mating, nesting, foraging, guarding and social interactions | 9 |
| Newly Emerged Male | < 36 hours | No | 12 |
| Aged Male (Lab) | 10 days | No | 10 |
| Wild Male | Minimum 3 weeks | Yes: Foraging, scent collection, male-male interactions, perfume display and potential mating, | 13 |

**Table 1.** Summary of all individuals included in the study, organized by sex, age class and experience. “Newly emerged” individuals were collected at soon after eclosion and prior to any social interactions or flight. “Aged” individuals were maintained under laboratory conditions deprived from foraging, social interactions, and flight. “Guard” and “dominant” females were collected from natural nest after behavioral monitoring. The asterisks indicate that age class are informed estimate based on filed observations rather than precise aged. “Wild males” were free-flying individuals with a minimum estimate of three weeks of experience based on amount of perfume extracted from their hind-legs during collection. Sample sizes (n) are provided for each group.
