# Supplementary figures and images for: Sex-Specific Neuroplasticity in the Brain of a Facultatively Social Orchid Bee
Source: Integr Comp Biol. 2026 Mar 23;66:icag012. doi: 10.1093/icb/icag012 (PMC13069684; doi:10.1093/icb/icag012)

## Slide 1
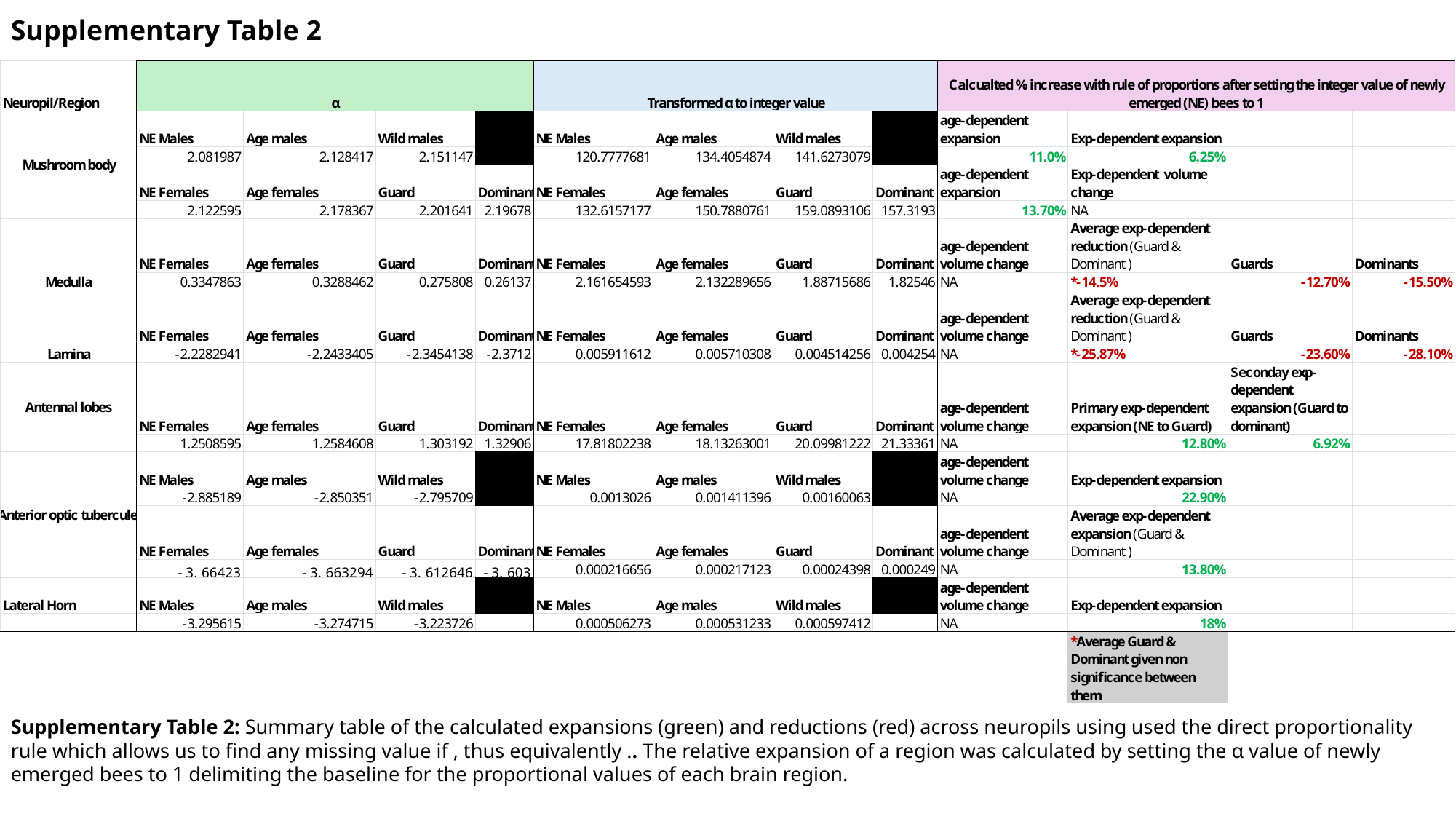

Supplementary Table 2

Supplement: icag012_Supplemental_Files [file icag012_supplemental_files.zip › icb-2026-0003-File009.pptx]
